# Patient-reported outcomes in meta-analyses --Part 2: methods for improving interpretability for decision-makers

**DOI:** 10.1186/1477-7525-11-211

**Published:** 2013-12-21

**Authors:** Bradley C Johnston, Donald L Patrick, Kristian Thorlund, Jason W Busse, Bruno R da Costa, Holger J Schünemann, Gordon H Guyatt

**Affiliations:** 1 Department of Anesthesia and Pain Medicine, and Institute of Health Policy, Management and Evaluation, University of Toronto, Toronto, ON, Canada; 2 Child Health Evaluative Sciences, Hospital for Sick Children Research Institute, Toronto, ON, Canada; 3 Department of Health Services, University of Washington, Seattle, WA, USA; 4 Seattle Quality of Life Group, Seattle, WA, USA; 5 Department of Clinical Epidemiology and Biostatistics, McMaster University, Hamilton, ON, Canada; 6 Department of Anesthesia, McMaster University, Hamilton, ON, Canada; 7 Division of Clinical Epidemiology & Biostatistics, Institute of Social and Preventive Medicine, University of Bern, Bern, Switzerland; 8 Department of Medicine, McMaster University, Hamilton, ON, Canada; 9 The Michael G. DeGroote Institute for Pain Research and Care, McMaster University, Hamilton, Canada

## Abstract

Systematic reviews and meta-analyses of randomized trials that include patient-reported outcomes (PROs) often provide crucial information for patients, clinicians and policy-makers facing challenging health care decisions. Based on emerging methods, guidance on improving the interpretability of meta-analysis of patient-reported outcomes, typically continuous in nature, is likely to enhance decision-making. The objective of this paper is to summarize approaches to enhancing the interpretability of pooled estimates of PROs in meta-analyses. When differences in PROs between groups are statistically significant, decision-makers must be able to interpret the magnitude of effect. This is challenging when, as is often the case, clinical trial investigators use different measurement instruments for the same construct within and between individual randomized trials. For such cases, in addition to pooling results as a standardized mean difference, we recommend that systematic review authors use other methods to present results such as relative (relative risk, odds ratio) or absolute (risk difference) dichotomized treatment effects, complimented by presentation in either: natural units (e.g. overall depression reduced by 2.4 points when measured on a 50-point Hamilton Rating Scale for Depression); minimal important difference units (e.g. where 1.0 unit represents the smallest difference in depression that patients, on average, perceive as important the depression score was 0.38 (95% CI 0.30 to 0.47) units less than the control group); or a ratio of means (e.g. where the mean in the treatment group is divided by the mean in the control group, the ratio of means is 1.27, representing a 27% relative reduction in the mean depression score).

## Introduction

Clinical trials evaluating medical treatments and health interventions increasingly incorporate self-reported measures from patients, often referred to as patient-reported outcomes (PROs). A PRO is defined as “any report of the status of a patient’s health condition that comes directly from the patient without interpretation of the patient’s response by a clinician or anyone else” [1]. Systematic reviews and meta-analyses of clinical trials often include PROs. In Part 1 of this series, we addressed the importance of PROs for health care decision-making, illustrated the key risk of bias issues that systematic reviews of PROs should consider and provided guidance on combining PROs in meta-analyses [2]. Part 1 used examples of PROs employed in assessing and summarizing post-operative pain and chronic obstructive pulmonary disease outcomes, while in this article, in addition to using examples of chronic obstructive pulmonary disease–we primarily use an example of summarizing PROs from clinical trials in depression. The structure of this article borrows from a recent article we published on preparing Summary of Findings tables for systematic reviews of continuous outcomes prepared by the Grading of Recommendations Assessment, Development and Evaluation (GRADE) working group [3]. Summary of Findings tables are an approach the GRADE working group developed for the presentation of findings particular to each outcome of interest in systematic reviews and meta-analysis. The tables have been developed for the presentation of continuous and dichotomous outcomes [3,4]. The purpose of this article is to summarize five presentation approaches to enhancing the interpretability of pooled estimates of PROs.

## Methods for improving the interpretability of pooled data

Meta-analyses of clinical trials routinely provide enough information for decision-makers to evaluate the extent to which chance can explain apparent differences between interventions. The interpretation of the magnitude of treatment effects is typically more challenging. First, if trials have used the same instrument, decision-makers may have difficulty interpreting the size of the effect. For instance, if told that the weighted mean difference between rehabilitation and standard care in a series of randomized trials using the Chronic Respiratory Questionnaire (CRQ) was 1.0 (95% CI 0.6-1.5), many readers would have no idea if this represents a trivial, small but important, moderate, or large effect [5-7].

The situation becomes even more challenging when trials use different instruments to measure the same or similar constructs. For instance, there are at least five instruments available to measure health-related quality of life (HRQoL) in patients with chronic obstructive respiratory disease (Chronic Respiratory Questionnaire, Clinical COPD Questionnaire, Pulmonary Functional Status and Dyspnea Questionnaire, Seattle Obstructive Lung Disease Questionnaire, St George’s Respiratory Questionnaire) [8]. We will deal with these two situations–all trials having used the same instrument, and trials having used different instruments–in turn.

### Summarizing a single PRO: beyond a mean difference and statistical significance

On occasion, individual studies using continuous variables will provide data that facilitate creating meaningful dichotomies. For example, studies of the impact of thrombolytic therapy after stroke typically use the Rankin instrument that classifies patients into one of six categories of disability from no symptoms to severe handicap requiring constant attention. Authors of a systematic review evaluating the impact of thrombolytic therapy in patients with stroke dichotomized the Rankin instrument, creating a “bad outcome” category of those dead, or moderately or severely disabled (which they labelled as “dependent”) and a “good outcome” category of those with no symptoms, no significant disability, slight disability or moderate disability [9]. The reviewers were therefore able to present results showing that thrombolytic therapy significantly reduced the proportion of patients who were dead or dependent at the end of 3 to 6 months of follow-up (OR 0.81, 95% CI 0.73 to 0.90). This presentation facilitates interpretation by the review’s readers. A priori, reviewers should choose and justify their threshold when dichotomizing PROs, and consider conducting sensitivity analyses providing results for reasonable and extreme thresholds to support a better understanding of the generalizability of the results.

When authors do not provide information that would facilitate meaningful dichotomies, the systematic reviewer can aid interpretation by reporting the range of possible results, and the range of means in treatment and control groups in the studies. Particularly useful–if it is available–is an estimate of the smallest difference that patients are likely to consider important (the minimally important difference or MID). There are a variety of methods for generating estimates of the MID [10,11], the application of which can lead to statements such as the following in a systematic review of the impact of respiratory rehabilitation in patients with chronic lung disease on HRQoL: “for each of the Chronic Respiratory Questionnaire domains (dyspnea, fatigue, emotional function and mastery), the common effect size exceeded the MID (0.5 point on the 7-point scale).” Authors also reported that for each of the Chronic Respiratory Questionnaire domains, the lower limit of the confidence interval around the common treatment effect exceeded the MID (e.g. dyspnea domain: 1.0; 95% CI 0.8-1.2) [12].

Although this is very helpful, it potentially tempts clinicians to make inappropriate inferences. If the MID is 0.5 and the mean difference between treatments is 0.4, clinicians may infer that nobody benefits from the intervention; if the mean difference is 0.6, they may conclude that everyone benefits. Both inferences are a misinterpretation as they ignore the distribution of benefit between individuals. We suggest the following guide for interpretation given a 0.5 MID: if the pooled estimate is greater than 0.5, and one accepts that the estimate of effect is accurate, many patients may gain important benefits from treatment. If the estimate of effect lies between 0.25 and 0.5, the treatment may benefit an appreciable number of patients. As the pooled estimate falls below 0.25 (i.e. 50% of the MID), it becomes progressively less likely that an appreciable numbers of patients will achieve important benefits from treatment.

### More than one PRO: beyond a standardized mean difference & statistical significance

As the prior discussion pointed out, when pooling across different PROs that measure a common construct the weighted mean difference is much more challenging to generate and we therefore replace it with a unitless measure of effect called the standardized mean difference (SMD) or “effect size”. This involves dividing the difference between the intervention and control means in each trial (i.e., the mean difference) by the estimated between-person standard deviation (SD) for that trial [13]. The SMD expresses the intervention effect in SD units rather than the original units of measurement, with the value of a SMD depending on both the size of the effect (the difference between means) and the SD of the outcomes (the inherent variability among participants). This approach has a number of limitations. First, decision-makers will not have an intuitive sense of the importance of the effect on the basis of the SD unit report. Second, it has statistical limitations (the same effect will appear different if population heterogeneity across eligible trials differs) [14].

Unfortunately, there is no fully satisfactory way of providing a sense of the magnitude of effect for a PRO when one has had to resort to effect sizes to generate a summary estimate. One can offer readers standard rules of thumb in interpretation of effect sizes (for instance 0.2 represents a small effect, 0.5 a moderate effect, and 0.8 a large effect [15] or some variation (for instance, <0.40 = small, 0.40 to 0.70 = moderate, >0.70 = large). However, effect size interpretations are often disease-specific and context-specific, further warranting an explanation for the reader. Another, perhaps even less satisfactory approach suggests that a standardized mean difference of 0.5 approximates, in many cases, the MID [16,17]. It is, however, very unlikely that a single SD ratio (explained below) will apply to all instruments.

When at least one instrument has an established anchor-based MID, the MID to SD ratio (SD ratio) may provide an estimate of MID values for instruments without an established MID. For a given PRO instrument, the SD ratio is the anchor-based MID divided by the baseline SD (or, if not reported, the end-of-treatment SD for the control group). When several SDs are available from a number of trials, a median SD ratio can be computed, and can be used to estimate the MID for a PRO instrument for which an anchor-based MID is not established. This is done by multiplying the SD by the median SD ratio [18]. This method assumes that the SD ratio is relatively constant across a range of PRO instruments measuring the same or similar constructs (e.g. disease-specific quality of life) in similar populations. For instance, the SD ratios based on the four instruments with known anchor-based MIDs were 0.26 (St George’s Respiratory Questionnaire), 0.51 (Chronic Respiratory Questionnaire), 0.34 (Montgomery Asberg Depression Rating Scale (MADRS)) and 0.86 (17-item Hamilton Rating Scale for Depression (HRSD)). These findings suggest that a single SD ratio based on average ratios between MIDs and baseline SDs is very unlikely to apply to all instruments and advocate the need for sensitivity analyses to explore the extent to which pooled estimates are robust to a variety of MID estimates [18].

Many authors have proposed alternatives to the SMD that produce summary estimates that clinicians can interpret more easily, some of which rely on standard deviations being similar across trials, and some of which do not [14,18-22]. Thus far, alternatives to the SMD have seen limited use and few studies have compared the SMD approach to the available alternatives [14,19,23-26].

Despite their limited use, the alternative approaches to summarizing results to enhance interpretability can be very useful. Consider, for instance, a systematic review assessing paroxetine vs placebo for the treatment of major depression in adults, which included 34 randomized trials employing the HRSD (n = 30) and the MADRS (n = 4) [27]. The MADRS ratings can be added to form an overall score ranging from 0 to 60; whereas for the HRSD, a number of versions exist, the most common being the 17-item HRSD, with overall scores ranging from 0 to 50 [28]. The majority of the included trials employing the HRSD used the 17-item version. Investigators have established 3 as the anchor-based MID for the 60-point Montgomery Asberg Depression Rating Scale [29] and 7 as the anchor-based MID for the 50-point Hamilton Rating Scale for Depression [30]. Providing pooled estimates of effect and making results interpretable for decision-makers mandates use of one of five available presentation approaches that we will summarize here, the merits of which–and our associated recommendations–are presented in Table 1. The five presentation approaches discussed are: standard deviation units (i.e. the standardized mean difference); conversion to the natural units of the most common instrument; conversion to dichotomized relative and absolute effects; ratio of means; and minimally important difference units.

**Table 1:** Five approaches to presenting pooled PRO variables when primary studies have used different instruments to measure the same construct

**Approach**	**Description**	**Advantages**	**Disadvantages**	**Recommendation**
(A) Standard deviation (SD) units (standardized mean difference; effect size)	The pooled mean difference is presented in standard deviation units	(+) Widely used	(-) Interpretation challenging	Consider complimenting other approaches with this; it is not recommended to use this approach independently.
			(-) Misleading when trial SDs are heterogeneous	
(B) Natural units	Linear transformation of trial data to most familiar scale	(+) Easier to interpret if scale well-known	(-) Few instruments in clinical practice are easy to interpret	Approaches to conversion to natural units include those based on SD units and re-scaling approaches. We suggest the latter. In rare situations when instrument very familiar to front line clinicians seriously consider this presentation
(C) Relative and absolute dichotomized effects	Obtain proportion above threshold in both groups and calculate relative or absolute binary effect measure	(+) Very familiar to clinical audiences	(-) Involve statistical assumptions that may be questionable	If the minimal important difference is known use this strategy in preference to relying on SD units
				Always seriously consider this option
(D) Ratio of means	The ratio between the mean responses in the intervention and control group	(+) May be easily interpretable to clinical audience	(-) Not applicable for change scores	Consider as complementing other approaches, particularly the presentation of relative and absolute effects
		(+) Fewer questionable assumptions	(-) Interpretation requires knowledge of control group mean	
(E) Minimal important difference units	The pooled mean differences is presented in MID units	(+) May be easily interpretable to clinical audience	(-) Only applicable when minimally important difference is known	Consider as complementing other approaches, particularly the presentation of relative and absolute effects

## Presentation approaches

### Standard deviation units - standardized mean difference

One way of generating a pooled estimate when trials have measured the same construct with different instruments is to divide the difference between the intervention and control means (i.e., the difference in means) in each trial by the estimated between-person standard deviation (SD) (see row A in Table 2[13]. This measure is often referred to as the standardized mean difference (SMD) or Cohen’s effect size.

**Table 2:** Application of summary approaches to paroxetine vs placebo for major depression in adults

**Outcomes**	**Estimated risk with Placebo**	**Absolute reduction in risk with Paroxetine**	**Relative effect (95% CI)**	**Number of participants (studies)**	**Confidence in effect estimate**^**1**^	**Comments**
**(A) Standard deviation units**	The depression score in the paroxetine groups was on average 0.31 SDs (0.24 to 0.38 lower than in the placebo groups)		---	5736 (34)	**⊕⊕**OO^2,3^ low	As a rule of thumb, 0.2 SD represents a small difference, 0.5 moderate, and 0.8 large (Cohen, 1988)

**(B) Natural units**						
Major depression measured on Hamilton Rating Scale for Depression, generally scored from 0 to 50, higher scores indicate more severe depression	The mean depression scores with placebo ranged from 3.1 to 11.3	The mean depression score in the intervention groups was on average 2.47 (1.91 to 3.03) lower		5736 (34)	**⊕⊕**OO^2,3^ low	Scores estimated based on an SMD of 0.31 (95% CI 0.24 to 0.38)The minimal important difference on the 0 to 50 depression scale is 7 points. Although the depression score was on average only 2.47 lower, the corresponding NNT is 11
**(C) Risk difference**	50 per 100 patients	39 per 100 patients	OR=1.64 (95% CI 1.47 to 1.84)	5736 (34)	**⊕⊕**OO^2,3^ low	This approach uses binomial and equal variance assumptions and baseline risks, and demonstrates that for every 100 patients treated with paroxetine, 11 will achieve important improvement
		Differences in proportion achieving important improvement				
		0.11 (95% CI 0.07 to 0.16) in favor of paroxetine				
**(D) Ratio of means**	---	---	Ratio of means	5736 (34)	**⊕⊕**OO^2,3^ low	Weighted average of the mean depression score in paroxetine group divided by mean depression score in placebo. RoM method provides similar effect estimates compared with the traditionally used standard deviation unit, with SMDs of 0.2, 0.5, and 0.8, corresponding to increases in RoM of approximately 8%, 22%, and 37%, respectively (Friedrich 2011).
			1.27 (1.18 to 1.36)			
**(E) Minimal important difference units**	The depression score in the paroxetine groups was on average 0.38 (95% CI 0.30 to 0.47) minimal important difference units less than the control group		---	5736 (34)	**⊕⊕**OO^2,3^ low	An effect less than half the minimal important difference suggests a small effect


Note: Investigators measured depression using different instruments, higher scores indicate more severe depression. ^1^Quality rating from 1 (very low quality) to 4 (high quality); ^2^Evidence limited by heterogeneity between studies; ^3^Evidence limited by risk of bias (i.e. missing participant data and potential for selective reporting bias).

Presenting results in SD units (as an SMD) is by far the longest standing and most widely used approach and is recommended in the Cochrane Handbook [13]. Calculating and presenting results in SD units has, however, major limitations. First, clinicians and their patients are unlikely to be able to relate to this way of presenting results [26]. Second, if the variability or heterogeneity in the severity of patients’ condition (and thus the variability in scores on the chosen outcome) varies between trials, the SDs will also vary. As a result, trials that enrol heterogeneous groups of patients will yield smaller SMDs than trials enrolling less heterogeneous patients, even if the actual (not standardized) mean difference estimates–and thus the absolute estimate of the magnitude of treatment effect–is similar across all trials. Finally, if very homogenous populations are enrolled, SD units can give a misleading, inflated impression of the magnitude of treatment effect.

In Table 2, the presentation in SD units suggest a small treatment effect. The structure of the Summary of Findings table, however, is not well suited to this presentation. If authors use the SMD, it is not sensible to present absolute values in the intervention and comparison groups because studies have used different measurement instruments with different units. One approach to this dilemma, presented in Table 2, is to present the SMD in place of the two columns usually devoted to absolute rates. An alternative is to present the median value from the studies that used the most familiar measure of the concept in the control group column, and the SMD in the intervention group column. To aid interpretability of a metric unfamiliar to clinicians or patients, a comment provides a rule-of-thumb guide to the significance of various effect sizes [15] (see row A, Table 2).

### Conversion into units of the most commonly used instrument

A second approach (see row B in Table 2) converts the effect size back into the natural units of the outcome measure most familiar to the target audience(s). There are two statistical approaches to making the conversion. One calculates the absolute difference in means by multiplying the SMD by an estimate of the SD associated with the most familiar instrument. For example, one might assume that the HRSD, a 0 to 50 point measure with evidence of reliability and validity, is the most familiar depression instrument among decision-makers [31]. In this case the magnitude of effect for the chosen instrument is 2.47. This result would be of limited use without knowledge of the MID, and thus the comment includes the estimated MID (7 points) [30], suggesting a small, and perhaps very small, effect (row B, Table 2).

The other statistical approach makes a simple conversion–before pooling and without calculating the SMD–of other instruments to the units of the most familiar instrument [25]. In this case, we chose the Hamilton Rating Scale for Depression, and re-scaled the mean and SD of the other instruments to HRSD units. Given the MID of the HRSD (7 units), the mean difference in change of 2.50 suggests a small treatment effect of paroxetine [30].

This second approach, presenting in units of the most familiar instrument, may be the most desirable when the target audience(s) have extensive experience with that instrument, particularly if the MID is well established [10]. Nevertheless, the natural unit presentation may, in relation to the MID, still be misleading. In this case, the absolute difference is less than half the MID. This may lead clinicians to conclude the effect of treatment is trivial. While it is correct that the effect is small, as indicated above, it may still be important. For instance, a mean difference of 2.50 units in the HRSD (in which the MID is 7.0) is translated into a difference of the proportion of patients benefiting in experimental and control groups of 9.2%, and thus a number needed to treat (NNT) of approximately 11.

### Conversion to dichotomized relative and absolute effects

A third approach (see row C in Table 2) converts the continuous measure into a dichotomy and thus allows calculation of relative and absolute effects on a binary scale. One method to generate a dichotomy from continuous data relies on the SMD and assumes that results of both treatment and control group are normally distributed and have equal variances [21,32]. Meta-analysts usually make these assumptions when they calculate SMDs [21]. We have used this approach in Table 2, row C, and it suggests a small relative effect and a small but still potentially important absolute effect. This approach has the advantage that you can apply it easily by consulting Tables 3 and 4, which provides the relation between the SMD, control group response rate, and the resulting risk difference. Table 3 presents the conversion when the outcome is undesirable (e.g. depression) and Table 4 when the outcome is desirable (e.g. response to treatment).

**Table 3:** For situations in which the event is undesirable, reduction [or increase if intervention harmful] in adverse events with the intervention

**Control group response rate**	**0.1**	**0.2**	**0.3**	**0.4**	**0.5**	**0.6**	**0.7**	**0.8**	**0.9**
SMD = -0.2	-0.03	-0.05	-0.07	-0.08	-0.08	-0.08	-0.07	-0.06	-0.040
SMD = -0.5	-0.06	-0.11	-0.15	-0.17	-0.19	-0.20	-0.20	-0.17	-0.12
SMD = -0.8	-0.08	-0.15	-0.21	-0.25	-0.29	-0.31	-0.31	-0.28	-0.22
SMD = -1.0	-0.09	-0.17	-0.24	-0.23	-0.34	-0.37	-0.38	-0.36	-0.29

**Table 4:** For situations in which the event is desirable, increase [or decrease if intervention harmful] in positive responses to the intervention

**Control group response rate**	**0.1**	**0.2**	**0.3**	**0.4**	**0.5**	**0.6**	**0.7**	**0.8**	**0.9**
SMD = 0.2	0.04	0.61	0.07	0.08	0.08	0.08	0.07	0.05	0.03
SMD = 0.5	0.12	0.17	0.19	0.20	0.19	0.17	0.15	0.11	0.06
SMD = 0.8	0.22	0.28	0.31	0.31	0.29	0.25	0.21	0.15	0.08
SMD = 1.0	0.29	0.36	0.38	0.38	0.34	0.30	0.24	0.17	0.09

This approach suffers from three important limitations. First, the dichotomous outcome that the intervention is decreasing is often not self-evident from the continuous outcome from which it is derived. We obtain a difference in the proportion of patients in intervention and control groups above some threshold, but the choice of that threshold is often arbitrary. In this example (Table 2, row C), fortunately, we can specify the threshold as an important improvement in depression (i.e. a change of 1 MID or more is representative of a 7 point difference on the HRSD). Second, the method requires investigators to specify the proportion of control patients with an improvement of at least one MID. Choosing this proportion may also be difficult. For instance, if one knows that control group depression scores varied from 23 to 44, with standard deviations around 12, how is one to decide the proportion of patients who failed to experience an important improvement with placebo? One possible approach would be, as a first step, to convert the mean value of the PRO in the control group into proportion of patients experiencing an improvement of at least one MID, for each of the studies included in the meta-analysis [32]. Reviewers could then use the median proportion across all studies for the conversion of the overall SMD [21]. The latter problem is ameliorated to some extent because only at the extremes of control proportions do the proportions benefiting change substantially. A third limitation is that the approach, by relying on the SMD, is vulnerable to whether study populations had very similar scores on the outcome of interest, or whether scores were widely variable.

Other statistical approaches also rely on the SMD to generate dichotomous presentations for continuous outcomes [22,33]. They share similar limitations, with the exception that they do not require specification of the control group response rate, and one approach becomes unstable when the underlying control group response rate is less than 20% or greater than 80% [22].

Another strategy for creating dichotomies and generating estimates of relative and absolute effect relies on knowledge of the MID. In applying the approach, we assume normal distributions of data and then calculate the proportions of participants in the intervention and control groups in each study that demonstrated an improvement greater than the MID [25]. The results are then pooled across studies. Applying this approach in Table 2, findings suggest small to moderate relative and absolute benefit in depression as a result of paroxetine therapy (Odds Ratio 1.64; 95% CI 1.47 to 1.84; Risk Difference 0.11; 95% CI 0.07 to 0.16, in favor of paroxetine).

If one only has post-test data (rather than magnitude of change), one can apply this approach if evidence exists regarding meaningful thresholds. For instance, if one knows that people with scores of less than 8 on the HRSD are considered to be not depressed, one could examine the proportion of individuals below that threshold.

If such meaningful thresholds do not exist, one can still use post-test data if one assumes that the minimally important change within an individual corresponds, on average, to the minimally important difference between individuals. Making this assumption, one can calculate the difference in the proportion who benefit in intervention and control. To do this, one takes the mean value in the control group plus one MID unit, and calculates the proportion of patients in each group above that threshold.

### Ratio of means

A fourth approach (see row D in Table 2) may appeal to clinicians: calculate a ratio of means (RoM) between the intervention and control groups [20]. Advantages of RoM include the ability to pool studies with outcomes expressed in different units, avoiding the vulnerability of heterogeneous populations that limits approaches that rely on SD units, and ease of clinical interpretation. However, a limitation of this RoM method is that it is designed for post-test scores only.

It is possible to calculate a ratio of change score if both intervention and control groups change in the same direction in each relevant study, and this ratio may sometimes be informative. Limitations include: i) the unlikelihood of intervention and control group changes in the same direction in all studies and ii) the possibility of misleading results if the control group change is very small–in which case, even a modest change in the intervention group will yield a large and therefore misleading ratio of mean changes.

In the paroxetine for depression example (Table 2), the ratio of means approach suggests a 27% increase in the mean depression score–meaning that those receiving paroxetine have a 27% decrease in depression manifestations relative to the placebo control group, an effect that strikes us as moderate and important.

### Minimally important difference units

A final strategy pools across studies in the same way as the SMD, but instead of dividing the mean difference of each study by its SD, it divides by the MID associated with that outcome [14]. The final output, instead of being in SD units, is in MID units. This approach avoids the problem of varying SDs across studies that may distort estimates of effect in approaches that rely on the SMD. It may, in addition, be more easily interpretable though it risks the possibility that a difference less than the MID may be interpreted as trivial when a substantial proportion of patients have achieved an important benefit. This is almost certainly an inaccurate interpretation, as conversion into an absolute risk difference and NNT would demonstrate (in this case a risk difference of 0.11 equates to an NNT of 9). In addition, to the extent that the MID estimate is not based on secure evidence, the approach becomes more questionable [18]. As stated in the comment in Table 2 (row E), the result for paroxetine for depression is an effect less than half of one MID, suggesting a small treatment effect. We suggest the following guide for interpretation: if the pooled estimate is greater than 1 MID unit, many patients are likely to gain important benefits from treatment. If the estimate of effect lies between 0.5 and 1 MID unit, the treatment may benefit an appreciable number of patients. As the pooled estimate falls below 0.5 MID units it becomes progressively less likely that an appreciable numbers of patients will achieve important benefits from treatment.

#### *Natural frequencies and numbers needed to treat*

A systematic review of the literature suggests that natural frequencies (× of 100 people not taking any osteoporotic drug will suffer a hip fracture over a three year period) optimizes understanding for most patients and health professionals [34]. Another approach for readers who are familiar with the measure is to present the NNT (the inverse of the proportion benefiting) [23]. Any approach that yields a proportion can be converted to NNTs. Furukawa offers an approach based on the binomial and equal variance assumptions, which meta-analysts usually presuppose when they resort to standardized mean differences [21,35]. Tables 3 and 4 shows the results of this method, which provides the relation between the SMD, control group response rate, and the resulting risk difference. Table 3 presents the conversion when the outcome is undesirable (e.g. depression) and Table 4 when the outcome is desirable (e.g. response to treatment). The NNT can be derived from the inverse of the risk difference.

#### *Summary and recommendations for enhancing interpretation*

We have provided an overview of available methods, including the strengths and limitations of the approaches, for improving the interpretability of pooled estimates of PROs when trials measure effects using the same instrument as well as a diversity of instruments. A complete summary of strengths and limitations of each of the methods for pooling diverse instruments, including details of the underlying statistical assumptions and methods, is available in an earlier review [25].

When trials all use the same PRO it is important to report results beyond a mean difference and statistical significance as suggested above. When primary studies have employed more than one instrument it will almost certainly be informative to report one or more alternatives to the SMD. Calculation and reporting of several approaches will, if the estimate of effect is of apparently similar magnitude, be reassuring (and if they are not, will present a challenge that reviewers should address). Of the two approaches for converting to natural units of the most familiar instrument, we recommend re-scaling the observed means and SDs in the intervention and control groups over multiplying the SMD by an estimate of the SD associated with the most familiar instrument because the former approach does not depend on similarity of patient heterogeneity between studies.

Because of its familiarity to clinicians, in most instances reviewers might choose to present one of the measures that generates relative and/or absolute measures of effect. Of these approaches, if all instruments have an established MID, we recommend presenting results as a risk difference with corresponding risks, presented as a natural frequency, in the experimental group and control group as illustrated in Table 2. Consideration of the relative advantages and disadvantages of each presentation method when pooling PRO data will help ensure that data is interpretable to patients, clinicians and other key decision-makers in the health care domain.

## Abbreviations

CRQ: Chronic respiratory questionnaire; HRSD: Hamilton rating scale for depression; HRQoL: Health-related quality of life; MID: Minimal important difference; MADRS: Montgomery Asberg depression rating scale; SMD: Standardized mean difference.

## Competing interests

The authors declare that they have no competing interests.

## Authors’ contributions

BCJ: concept, data analysis, interpretation of data, manuscript drafting, preparation and approval, administrative support. DLP: concept, interpretation of data, manuscript preparation and approval. KT: concept, data analysis, manuscript preparation and approval. JWB: interpretation of data, manuscript preparation and approval. BRDC: interpretation of data, manuscript preparation and approval. HJS: interpretation of data, manuscript preparation and approval. GHG: concept, data analysis, interpretation of data, manuscript preparation and approval. All authors read and approved the final manuscript.
